# Systematic review and stratified meta-analysis of the efficacy of carnosine in animal models of ischemic stroke

**DOI:** 10.1177/0271678X16658302

**Published:** 2016-07-08

**Authors:** Charles K Davis, Peter J Laud, Zsanett Bahor, GK Rajanikant, Arshad Majid

**Affiliations:** 1 School of Biotechnology, National Institute of Technology Calicut, Calicut, India; 2 Statistical Services Unit, University of Sheffield, Sheffield, UK; 3 Centre for Clinical Brain Sciences, The University of Edinburgh, Edinburgh, UK; 4 Sheffield Institute for Translational Neuroscience (SITraN), University of Sheffield, Sheffield, UK

**Keywords:** Carnosine, ischemic stroke, meta-analysis, neuroprotection, systematic review

## Abstract

Carnosine is a naturally occurring pleotropic dipeptide which influences multiple deleterious mechanisms that are activated during stroke. Numerous published studies have reported that carnosine has robust efficacy in ischemic stroke models. To further evaluate these data, we have conducted a systematic review and meta-analysis of published studies. We included publications describing in vivo models of ischemic stroke where the neuroprotective efficacy of carnosine was being evaluated through the reporting of infarct volume and/or neurological score as outcomes. Overall efficacy was evaluated using weighted mean difference random effects meta-analysis. We also evaluated for study quality and publication bias. We identified eight publications that met our inclusion criteria describing a total of 29 comparisons and 454 animals. Overall methodological quality of studies was moderate (median = 4/9). Carnosine reduced infarct volume by 29.4% (95% confidence interval (CI), 24.0% to 34.9%; 29 comparisons). A clear dose-response effect was observed, and efficacy was reduced when carnosine was administered more than 6 h after ischemia. Our findings suggest that carnosine administered before or after the onset of ischemia exhibits robust efficacy in experimental ischemic stroke. However, the methodological quality of some of the studies was low and testing occurred only in healthy young male animals.

## Introduction

Over 15 million people suffer a stroke every year worldwide causing nearly six million deaths and leaving another five million people permanently disabled. The only approved drug for acute stroke is tissue plasminogen activator (tPA) but this drug has a short therapeutic time window of 4.5 h which limits the number of patients that are eligible to receive it.^1,2^ An urgent need exists for safe and effective therapies.^2^

Despite promising preclinical studies, no drugs have shown efficacy in clinical trials.^2,3^ One major reason for this is that most previous strategies have focused on modifying a single pathway or category of injury, whereas ischemia leads to a complex cascade of numerous biochemical and molecular events, ultimately causing the loss of cellular integrity and tissue destruction.^2^ It is highly desirable, therefore, to develop therapies which influence multiple pathways. Another reason that preclinical data have not translated to efficacy in clinical trials is that there has been poor experimental design or incomplete testing in the preclinical studies.^2,4^

Carnosine (beta-alanyl-L-histidine) is a naturally occurring dipeptide that is abundant in many animal tissues including brain.^5^ It has numerous properties that may be beneficial in limiting ischemia-induced brain injury. These include antioxidant, anti-excitotoxic, metal ion chelating, anti-matrix metalloproteinase, and intracellular pH buffering.^3,6–11^ All of these processes play an important role in the pathogenesis of brain infarction. Carnosine has been reported to be highly protective in both permanent and transient cerebral ischemia models by influencing multiple mechanisms including the attenuation of oxidative stress, apoptosis, and autophagy.^7,9,12^ An additional important property of carnosine is that it can cross the blood–brain barrier.^2^

However, to date, there has been no quantitative appraisal of the published data. Herein, we present a systematic review and meta-analysis of published studies using focal ischemia models of experimental stroke.

## Materials and methods

### Search strategy

We searched PubMed (1966–May 2016), Embase (1947–May 2016) and Web of Science (1900–May 2016) electronic databases using the search terms: (stroke OR (cerebral OR brain OR focal) AND (ischemia OR ischemic OR ischemia OR ischemic)) OR cerebrovascular OR middle cerebral artery OR MCA OR middle cerebral artery occlusion OR MCAO OR anterior cerebral artery OR ACA OR anterior cerebral artery occlusion OR ACAO OR experimental stroke AND (B-Alanyl-L-Histidine) OR (B-AlanylHistidine) OR (Beta-alanyl-L-histidine) OR (Bêta-Alanyl-L-Histidine) OR (Carnosina) OR (L-Carnosine) OR (N-Acetyl-Carnosine) OR (N-Acétyl-Carnosine) OR (N-Acetyl-L-Carnosine) OR (N-Acétyl-L-Carnosine) OR (beta-Alanyl-L-histidine) OR (Ignotine) OR (2-(3-aminopropanoylamino)-3-(3H-imidazol-4-yl)propanoic acid) OR (beta-ALA-HIS) OR (L-Histidine,.beta.-alanyl-) OR (L-alpha-ALANYL-L-HISTIDINE) OR (L-Histidine, N-.beta.-alanyl-) OR (2-(3-aminopropanoylamino)-3-(1H-imidazol-5-yl)propanoic acid) OR (2-(3-amino-propanoylamino)-3-(1H-imidazol-4-yl)-propionic acid)) NOT (liver OR kidney OR coronary OR myocardial OR testis OR testicular OR gastric OR mesenteric OR skeletal) limited to animals. Searches were limited to publications in English.

### Inclusion criteria

Two investigators (CKD and RGK) reviewed publications based on their titles and abstracts. Only studies evaluating the effect of carnosine in animal models using focal cerebral ischemia-induced by occlusion of the middle or anterior cerebral artery or their branches, compared with animal models receiving no carnosine were included in our review. Any route and dosage of carnosine administration at any time of delivery (before, during or after ischemia onset) were included. We chose infarct volume, as our primary outcome measure and neurobehavioral score as our secondary outcome measure.

### Quality assessment

Methodological quality of studies was assessed based on the modified CAMARADES study quality checklist comprising the following: (1) publication in a peer-reviewed journal, (2) statement of control of temperature, (3) randomization to treatment or control, (4) blinded induction of ischemia, (5) blinded assessment of outcome, (6) use of co-morbid animals, (7) sample size calculation, (8) statement of compliance with animal welfare regulations, and (9) statement regarding possible conflicts of interest.^13^ There was no exclusion based on study quality. The systematic review adopted preferred reporting items for systematic reviews and meta-analyses (PRISMA) guidelines.^14^

### Data extraction

From each study, we identified separate comparisons of our primary and secondary outcome measures quantified in a cohort of animal models of ischemic stroke where some animals had been given carnosine and control animals not given the treatment. We extracted data on species, sex, dosage range, time of administration, anesthetic used, type of ischemia, and route of drug delivery. The number of animals used, mean, and standard error of the outcome measure for treatment and control groups were also extracted. When multiple groups were served by a single control group, the weight of these animals in our analysis was adjusted by dividing by the number of treatment groups the control cohort served. Where multiple doses of a drug were given, the final aggregate outcome was taken, and the time of this outcome measure was taken as the time of assessment. When the data were only represented graphically or not mentioned at all in a paper, we contacted the authors to obtain raw data. In cases where there was no response from the authors, we either extracted the data from graphs published in their paper using the software Universal Desktop Ruler or where this was not possible we went on to exclude those studies. All data were extracted by two, non-blinded, reviewers (CKD and RGK).

### Data analysis

For infarct size, we calculated a normalized mean difference effect size for each comparison (normalized as a percentage of the mean in the control group) and combined these in a weighted mean difference meta-analysis using a random effects model.^15^ We used stratified meta-analysis to assess for the impact of drug dose; time of administration; type of ischemia; anesthetic used; species of animal used; and interval to quantification of outcome. The significance of differences between n groups was assessed by partitioning heterogeneity and by using the χ^2^ distribution with n−1 degrees of freedom (df). To allow for multiple comparisons, we set our significance level using Bonferroni correction to *p* < 0.01 and *p* < 0.007 for study quality and study characteristics variables, respectively. We assessed for publication bias using funnel plot.

## Results

**Figure 1.**
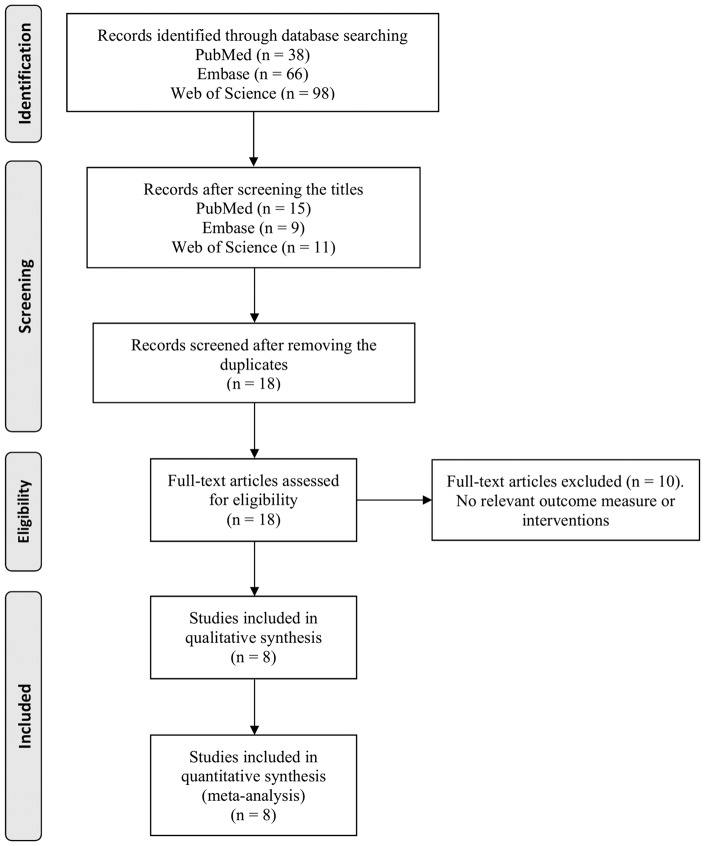
Flow diagram for studies included in this meta-analysis.

Our search of the literature identified 202 publications. After screening, only eight full articles were found to describe the effect of carnosine in animal models of focal cerebral ischemia and met our inclusion criteria.^7–11,16–18^Details of the review process are displayed in the PRISMA flow diagram (Figure 1). A summary of study characteristics of included publications are shown in Table 1. In total, studies included in our analysis reported data from 454 animals contributing to 29 comparisons and were done by 4 different research groups. All eight studies reported infarct size and four studies also reported a neurobehavioral score (127 animals in 7 comparisons). Only one study reported mortality data and therefore the influence of carnosine on mortality as an outcome measure was not analyzed. Only one study reported data from transient middle cerebral artery ischemia (Table 1).^8^ The data for transgenic mice from Shen’s paper were excluded as it was beyond the scope of our study.^16^

**Table 1.** Study characteristics.

Author	Year	Drug	Species	Sex	Anesthetic	Route of delivery	Outcome measure(s)	Dose (mg/kg)	Time of admin (min)	Time of assessment (h)	Ischemia type (P/T)	N (C)	N (Rx)
Rajanikant	2007	Carnosine	C57BL/6 mice	M	Isoflurane	IP	Infarct volume	100	−30	24	P	10	10
								500	−30	24	P	10	10
								1000	30	24	P	10	10
								1000	−30	24	P	10	10
								2500	240	24	P	7	7
								2500	120	24	P	7	7
Min	2008	Carnosine	C57BL/6 mice	NR	Halothane	IP	Infarct volume and neurobehavioral score	1000	−30	24	P	7	7
								1000	−30	72	P	7	7
								1000	−30	168	P	7	7
Shen	2010	Carnosine	C57BL/6 Mice	M	Sodium pentobarbital	IP	Infract volume and neurobehavioral score	250	−30	24	P	5	5
								500	−30	24	P	5	5
								750	−30	24	P	5	5
Bae	2013^8^	Carnosine	Sprague-Dawley rats	M	Isoflurane	IV	Infarct volume and Neurobehavioral score	500	180	24	T (180)	14	15
								1000	180	24	T (180)	14	15
								2000	180	24	T (180)	14	15
								1000	540	24	T (540)	7	13
								1000	720	24	P	13	15
								1000	540	24	P	14	15
								1000	360	24	T (360)	13	14
								1000	360	24	P	13	15
								1000	180	24	P	14	15
Bae	2013^11^	Carnosine	Sprague-Dawley rats	M	Isoflurane	IV	Infarct volume	1000	−30	24	P	10	10
Wang	2013	Carnosine	Sprague-Dawley rats	M	Isoflurane	IP	Infarct volume and neurobehavioral score	500	−30	72	P	6	6
								750	−30	72	P	6	6
								1000	−30	72	P	6	6
Park	2014	Carnosine	Rat (strain not given)	NR	Isoflurane	IP	Infarct volume	100	−30	2	P	5	5
								250	−30	2	P	5	5
								500	−30	2	P	5	5
Baek	2014	Carnosine	Sprague-Dawley rats	M	Isoflurane	Lateral tail vein	Infarct volume	1000	360	24	P	14	16

IV: intravenous; IP: intraperitoneal; NR: not reported, number after T in ischemia type columns denotes the duration of transient ischemia; M: males; N: number; C: control; Rx: treatment; P: permanent; T: transient.

### Reported study quality

All eight studies included in our analysis were published in peer-reviewed journals. Only one study reported having performed a sample size calculation. None of the studies included a statement of a potential conflict of interest. Total reported study quality score for each paper included in our review is summarized in Table 2. Median reported study quality score was 4 (interquartile range, 3–6) for the eight papers.

**Table 2.** Study quality score.

Author	Year	1	2	3	4	5	6	7	8	9	Quality Score
Bae	2013^8^	X	X	X	X			X	X		6
Rajanikant	2007	X	X								2
Min	2008	X	X	X					X		4
Bae	2013^11^	X	X	X	X	X			X		6
Shen	2010	X	X						X		3
Park	2014	X	X						X		3
Wang	2013	X	X	X					X		4
Baek	2014	X	X	X	X	X			X		6

Sample sizes were generally small; the median number of animals per group was 10 in the control group (interquartile range, 7–14) and 13 in the treatment group (interquartile range, 7–15). Five studies reported that they randomly allocated animals to treatment group and control groups (63%); two studies reported blinding their assessment of outcome (25%); three studies reported masked induction of ischemia (38%); and all studies reported regulating the temperature of the animals during the induction of ischemia.

Seven studies reported compliance with animal welfare regulations.

### Efficacy

**Figure 2.**
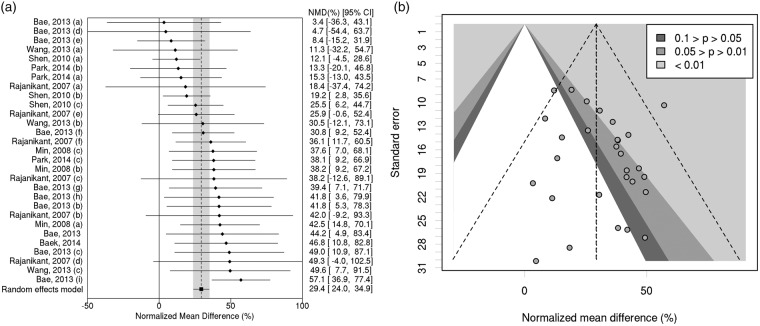
(a) Individual comparisons ranked according to magnitude of treatment effect on infarct volume. NMD: normalized mean difference. CI: confidence interval. (b) Contour-enhanced funnel plot showing standard error versus magnitude of treatment effect estimate. See supplementary material section, Table 1 for details of individual doses and times of administration in each study subdivision (a-i).

Results on infarct size for each comparison within the eight studies are summarized in supplementary Table 1. Carnosine treatment reduced infarct volume by 29.4% (95% CI, 24.0% to 34.9%; 29 comparisons; Figure 2(a)). Observed heterogeneity was low (χ^2 ^= 28.7, df = 28, *p* = 0.426). We did not observe any evidence of publication bias in the funnel plot (Figure 2(b)); Egger test *p* = 0.204).

**Figure 3.**
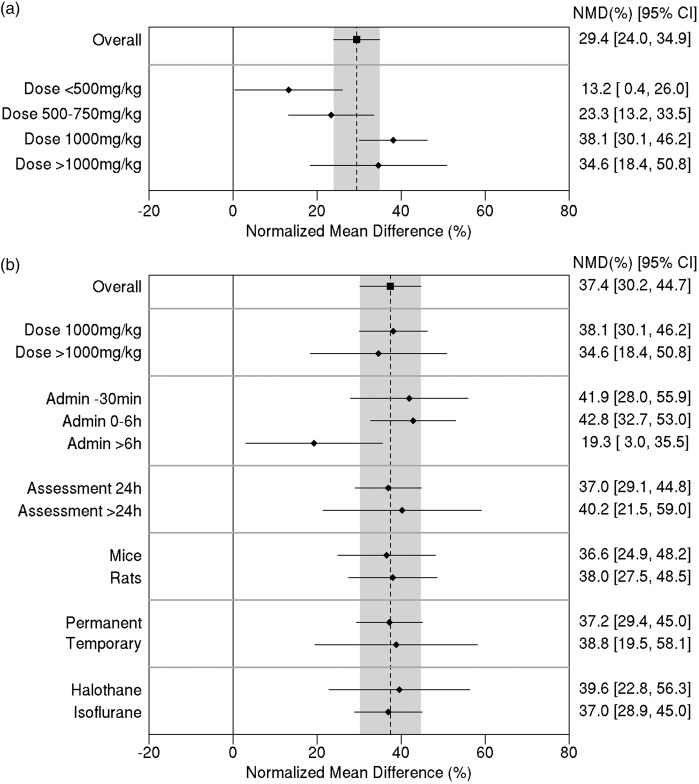
(a) Effect of carnosine dose on infarct volume. (b) Effect of study characteristic variables on the estimate of efficacy (for comparisons with carnosine dose ≥ 1000 mg/kg). The vertical grey bar represents the 95% confidence limits of the overall estimate. The horizontal error bars represent the 95% confidence intervals for the individual subgroup estimates. NMD: normalized mean difference. CI: confidence interval.

Stratified meta-analysis was done using the data for infarct volume. Initially, studies were grouped in terms of the carnosine dose (Figure 3(a)). Heterogeneity between dose subgroups was quantified as Q = 12.4, 3df, *p* = 0.006, demonstrating a clear difference in treatment effect by dose. There was a 38.1% reduction in infarct size (95% CI, 30.1% to 46.2%) for a dose of 1000 mg/kg compared with 23.3% (95% CI, 13.2% to 33.5%) for doses between 500 and 750 mg/kg and 13.2% (95% CI, 0.4% to 26.0%) for doses less than 500 mg/kg.

In the published data, carnosine dose was confounded with a number of other study characteristic variables. For example, out of the 11 comparisons involving lower doses (≤750 mg/kg), 10 of them administered the drug 30 min before the induction of ischemia. This makes it difficult to identify whether a difference in treatment effect in this portion of the data was associated with the dose or the time of administration.

In order to confirm the effect of dose independently from administration time, we looked at the dose effect for comparisons involving drug administration 30 min prior to the induction of ischemia only. The estimated effect of the 1000 mg/kg dose in this subset was similar to that in the full analysis. This suggests that the smaller effect estimates in the lower dose levels are indeed associated with the lower dose rather than the early administration time.

To avoid bias due to confounding factors such as dose, subsequent stratified meta-analysis was performed in an exploratory manner using only the 18 comparisons involving higher doses of carnosine (1000 mg/kg or more).

Looking at the effect of administration time, the between-subgroup heterogeneity was Q = 6.29; 2df; *p* = 0.0431. Allowing for multiple testing, this was not statistically significant. However, it is worth noting that the estimated reduction in infarct size was similar (42–43%) when carnosine was administered either 30 min prior to ischemia (95% CI, 28.0% to 55.9%) or within 6 h after ischemia (95% CI, 32.7% to 53.0%), whereas treatment more than 6 h after induction of ischemia was associated with only a 19% reduction (95% CI, 3.0% to 35.5%) in infarct size (Figure 3(b)).

Regarding the other potential effect modifiers, no significant between-subgroup heterogeneity was identified for study characteristics (Figure 3(b)). Nor was there any evidence of any relationship between effect size and any of the study quality parameters, including blinding of assessments (*p* = 0.53) and randomization (*p* = 0.62).

**Figure 4.**
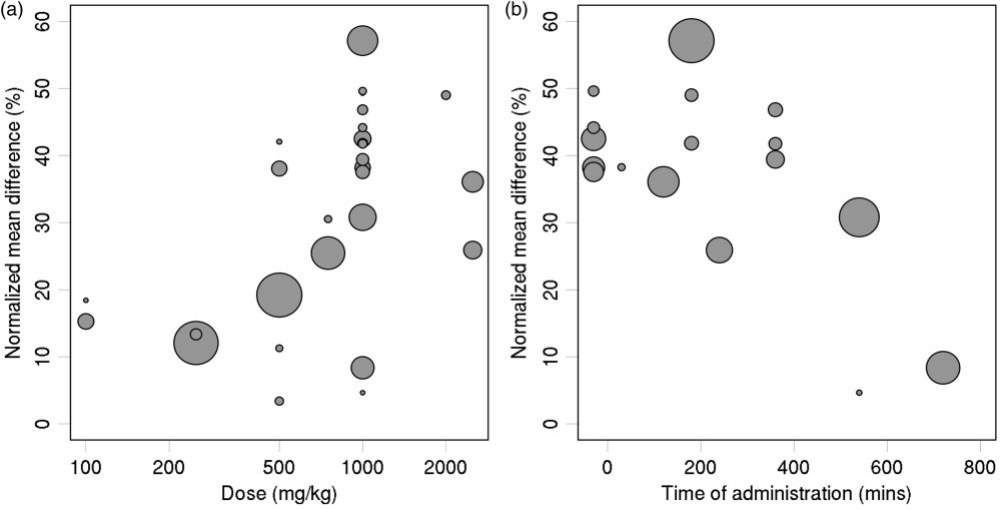
Treatment effect estimates versus: (a) carnosine dose, and (b) time of administration relative to ischemia (for comparisons with carnosine dose ≥ 1000 mg/kg). Sizes of plotted symbols represent precision of the estimated treatment effect (studies with larger sample size and/or less variability are represented by larger circles).

Bubble plots (Figure 4) were used to further illustrate the effects of dose (log scale) and administration time.

Stratified meta-analysis for neurobehavioral score was not performed due to insufficient number of comparisons.

## Discussion

To the best of our knowledge, this is the only systematic review and meta-analysis which has evaluated the efficacy of carnosine in animal models. Our analysis of data from eight stroke studies (454 animals), shows that carnosine has neuroprotective efficacy in reducing infarct size in animal models of stroke. We found that the dose of carnosine that was associated with the greatest efficacy was 1000 mg/kg. The observed treatment effects were larger in studies, where drug was administered immediately or up to six hours ( ≤ 6 h) after the induction of ischemia. However, some efficacy was even observed when carnosine was administered over 6 h after the induction of ischemia, albeit with a smaller treatment effect. This time window is clinically relevant and has translational implications as the median time for stroke patients to arrive at hospital is 4.3 h.^19^

### Study quality

Study quality overall was moderate. There were three studies with a quality score above five. Study quality criteria such as masked induction of ischemia, random allocation of animals to group, and blinded assessment of outcome were not commonly reported. However, there was no evidence of any relationship between effect size and any of the study quality parameters, including blinding of assessments and randomization. One explanation for this could be that quality parameters such as randomization and blinding did occur but were not reported in those publications.

One of the problems with systematic reviews is that not all studies that were carried out are published leading to publication bias. Research with statistically significant results is more likely to be submitted and published than work with non-significant results. We did not observe any evidence of publication bias in the funnel plot and the Egger test for significance.

### Relevance to clinical trials

Many interventions in the past that have shown efficacy in preclinical studies have then failed to exhibit efficacy in humans, possibly due to side effects, narrow therapeutic time windows, and targeting of single pathological pathways.^3,4^ Carnosine has a relatively long therapeutic time window in animals studies. It also favorably influences multiple pathways that are either activated or involved in the extent of tissue injury during ischemia such as excitoxicity, matrix metalloproteinase activity, endogenous anti-oxidant levels, free radical generation, pH buffering, and mitochondrial dysfunction.^2,7,8–11^ Taken together with the lack of reported side effects in human clinical studies in diabetes and heart failure, makes carnosine a very promising neuroprotective agent for acute stroke therapy.^20,21.^

## Limitations

One limitation to this systematic review is the small number of studies that met our inclusion criteria. The limited data available meant there were insufficient comparisons to carry out stratified meta-analysis for neurobehavioral score. Further, many of the studies were carried out by groups led by the same senior investigator and this may limit the generalizability of these results. Moreover, all the studies were carried out in healthy male animals without co-morbidity. To satisfy Stroke Academic Industry Roundtable (STAIR), future studies will need to test for efficacy and safety in aged animals, female animals and animals with co-morbidity such as hypertension. In addition, future studies will need to test efficacy and safety of co-administration with tPA.^22^

Despite searching for relevant studies in major electronic databases, we cannot rule out the possibility of missing studies.

Among the studies which used a dose of less than 1000 mg/kg, all but one of the comparisons administrated the drug 30 min before the induction of ischemia, which limits interpreting the effect of both time of administration and dose. Similar problems might have affected other study characteristics. For example, most of the low-dose studies used permanent ischemia, and a greater proportion of them used mice.

## Supplementary Material

Supplementary material
