# Supplementary material for: Systematic review and stratified meta-analysis of the efficacy of carnosine in animal models of ischemic stroke
Source: J Cereb Blood Flow Metab. 2016 Jul 8;36(10):1686–94. doi: 10.1177/0271678X16658302 (PMC5046161; doi:10.1177/0271678X16658302)
Supplement: Supplementary material [file Supplementary_Material_302.docx]

**Table I.** Summary results: Infarct volume

|  | Study | Dose (mg/kg) | Time of admin (mins) | N  (C) | Mean infarct volume in mm^3^(C) | SE  (C) | N  (Rx) | Mean infarct volume in mm^3^ (Rx) | SE (Rx) |
| --- | --- | --- | --- | --- | --- | --- | --- | --- | --- |
| (a) | Rajanikant 2007 | 100 | -30 | 10 | 14.0 | 1.67 | 10 | 11.4 | 2.15 |
| (b) | Rajanikant 2007 | 500 | -30 | 10 | 14.0 | 1.67 | 10 | 8.1 | 1.48 |
| (c) | Rajanikant 2007 | 1000 | 30 | 10 | 14.0 | 1.67 | 10 | 8.6 | 1.40 |
| (d) | Rajanikant 2007 | 1000 | -30 | 10 | 14.0 | 1.67 | 10 | 7.1 | 1.80 |
| (e) | Rajanikant 2007 | 2500 | 240 | 7 | 17.2 | 1.22 | 7 | 12.7 | 1.98 |
| (f) | Rajanikant 2007 | 2500 | 120 | 7 | 15.8 | 1.48 | 7 | 10.1 | 1.30 |
| (a) | Min 2008 | 1000 | -30 | 7 | 12.6 | 1.44 | 7 | 7.2 | 1.04 |
| (b) | Min 2008 | 1000 | -30 | 7 | 12.2 | 1.29 | 7 | 7.5 | 1.25 |
| (c) | Min 2008 | 1000 | -30 | 7 | 6.0 | 0.91 | 7 | 3.7 | 0.21 |
| (a) | Shen 2010 | 250 | -30 | 5 | 64.1 | 2.86 | 5 | 56.3 | 2.21 |
| (b) | Shen 2010 | 500 | -30 | 5 | 64.1 | 2.86 | 5 | 51.8 | 2.07 |
| (c) | Shen 2010 | 750 | -30 | 5 | 64.1 | 2.86 | 5 | 47.8 | 3.90 |
| (a) | Bae 2013(ref.8) | 500 | 180 | 14 | 50.2 | 4.70 | 15 | 48.5 | 6.10 |
| (b) | Bae 2013(ref.8) | 1000 | 180 | 14 | 50.2 | 4.70 | 15 | 29.2 | 4.60 |
| (c) | Bae 2013(ref.8) | 2000 | 180 | 14 | 50.2 | 4.70 | 15 | 25.6 | 5.40 |
| (d) | Bae 2013(ref.8) | 1000 | 540 | 7 | 38.7 | 9.10 | 13 | 36.9 | 7.30 |
| (e) | Bae 2013(ref.8) | 1000 | 720 | 13 | 63.3 | 3.80 | 15 | 58.0 | 6.60 |
| (f) | Bae 2013(ref.8) | 1000 | 540 | 14 | 60.1 | 2.80 | 15 | 41.6 | 6.00 |
| (g) | Bae 2013(ref.8) | 1000 | 360 | 13 | 44.4 | 5.70 | 14 | 26.9 | 4.60 |
| (h) | Bae 2013(ref.8) | 1000 | 360 | 13 | 44.3 | 6.00 | 15 | 25.8 | 6.20 |
| (i) | Bae 2013(ref.8) | 1000 | 180 | 14 | 59.7 | 2.80 | 15 | 25.6 | 5.50 |
|  | Bae 2013(ref.11) | 1000 | -30 | 10 | 47.1 | 7.60 | 10 | 26.3 | 5.60 |
| (a) | Wang 2013 | 500 | -30 | 6 | 25.0 | 2.82 | 6 | 22.2 | 2.63 |
| (b) | Wang 2013 | 750 | -30 | 6 | 25.0 | 2.82 | 6 | 17.4 | 2.39 |
| (c) | Wang 2013 | 1000 | -30 | 6 | 25.0 | 2.82 | 6 | 12.6 | 2.18 |
| (a) | Park 2014 | 100 | -30 | 5 | 201.0 | 15.42 | 5 | 170.3 | 8.41 |
| (b) | Park 2014 | 250 | -30 | 5 | 201.0 | 15.42 | 5 | 174.2 | 18.69 |
| (c) | Park 2014 | 500 | -30 | 5 | 201.0 | 15.42 | 5 | 124.5 | 8.88 |
|  | Baek 2014 | 1000 | 360 | 14 | 46.7 | 6.20 | 16 | 24.9 | 5.93 |

N: number; C: control; Rx: treatment SE: Standard error; ref: reference in the main document. The letters (a-i) correspond to the individual plots in Fig 2a.
